# The relationship between time to urgent or emergency pacemaker implantation and entry into residential aged care or death in older adults

**DOI:** 10.1007/s40520-026-03413-4

**Published:** 2026-06-07

**Authors:** Diane Nguyen, Madeleine Landwehr, Caitlyn Taylor, Eng Sim, Stephen Bacchi, Graham S. Hillis, Leon Flicker, Carl Schultz, Lucy Kilshaw, Nikola Stoyanov, Jamie W. Bellinge

**Affiliations:** 1 Department of Geriatrics, Royal Perth Hospital, Perth, Western Australia Australia; 2 Department of Psychiatry, Royal Perth Hospital, Perth, Western Australia Australia; 3 Faculty of Health and Medical Sciences, University of Adelaide, Adelaide, South Australia Australia; 4 Department of Cardiology, Royal Perth Hospital, Perth, Western Australia Australia; 5 Medical School, University of Western Australia, Perth, Western Australia Australia

**Keywords:** Pacemaker, Geriatric, Function, Residential aged care, Death

## Abstract

**Background:**

Older adults often require permanent pacemaker (PPM) insertion. Delays to PPM insertion may associate with poor health outcomes.

**Aims:**

To investigate the association between delayed PPM insertion and adverse outcomes in older adults.

**Methods:**

Patients aged ≥65 years who underwent non-elective inpatient PPM insertion at Royal Perth Hospital between April 2019 and January 2022 were included in this retrospective cohort study. The relationship between time from admission to PPM insertion (exposure) and death or residential aged care facility (RACF) admission was examined using Cox modelling.

**Results:**

In 375 patients, with a mean age of 80.8±7.5 years, the median [25th – 75th ] duration from admission to PPM insertion was 47 [22–90] hours. Time from admission to PPM insertion (days) was associated with an increased risk of death at 1635 [1048–1931] days follow-up in univariate regression models (HR 1.05, 95% CI [1.03–1.07], *p* < 0.001) and after adjusting for comorbidities, demographics, clinical presentation characteristics and number of inpatient complications (HR 1.03 [1.00–1.05], *p* = 0.027). Time from admission to PPM insertion was associated with an increased risk of RACF admission at 1789 [1546–2128] days follow-up in univariate (HR 1.03 [1.00–1.07], *p* = 0.049), but not in multivariable regression models (HR 1.00 [0.96–1.04], *p* = 0.92).

**Discussion:**

These findings support the existing literature associating delays to medical intervention and poor health outcomes in older adults.

**Conclusion:**

In older adults, prolonged time from admission to PPM insertion was associated with higher mortality. Future research is needed to determine if reducing time to PPM insertion may improve their health outcomes.

**Supplementary Information:**

The online version contains supplementary material available at 10.1007/s40520-026-03413-4.

## Introduction

Older adults are at a higher risk of bradyarrhythmia (abnormally slow heart rate), which may present acutely, require hospitalisation, and necessitate permanent pacemaker (PPM) insertion [1–3]. The primary objectives of PPM insertion are to improve prognosis and quality of life, and in older adults, the latter is closely related to the preservation of independence [4]. However, PPM insertion may not definitively improve prognosis [3] and hospitalisation and procedural intervention may increase the risk of loss of independence [5, 6]. In older adults undergoing PPM insertion, an improved understanding of factors contributing to death or loss of independence is required.

PPM insertion requires availability of an operating theatre or catheter laboratory, specialist team, adequate resourcing, and management of some patient-specific comorbidities, for example active infection, prior to the procedure. Variation in these factors may contribute to delayed PPM insertion, resulting in prolonged bed rest, deconditioning and risks of in-hospital complications, such as delirium, falls, or infection, thus increasing length of stay [7–9]. In patients admitted to surgical and medical wards, procedural delays are associated with increased risk of poor functional outcomes, mortality and residential aged care facility (RACF) admission [10–12]. PPM‑specific procedural delays are associated with an increased risk of acute adverse events in the inpatient setting [13]. These may be related to temporary transvenous pacing or infections such as urinary tract infections and pneumonia. Risks may be amplified in older adults, and the downstream consequences of adverse events, coupled with risks of deconditioning, may contribute to an increased risk of adverse long‑term outcomes. Therefore, we hypothesise that risks of poor functional outcomes, specifically death and RACF admission, are associated with PPM-specific procedural delays in older adults.

We aimed to examine the relationship between time from hospital admission to PPM insertions and subsequent RACF admission or death, in older adults needing urgent or emergent PPM insertion. We also aimed to examine the relationship between delayed PPM insertion and adverse in-hospital events.

## Methods

### Design and cohort

This retrospective cohort study included patients aged ≥65 years who underwent non-elective inpatient permanent pacemaker insertion at Royal Perth Hospital (RPH), Perth, Western Australia (WA), between April 2019 and January 2022. RPH is a tertiary hospital that provides on-call pacemaker services daily. Inpatient PPM insertion almost exclusively occurs Monday to Friday, though for emergency reasons can be performed on Saturday and Sunday.

Potentially eligible subjects were identified using the Australian Classification of Health Interventions (ACHI) code 38353-00, representative of PPM insertion, obtained from hospital administrative discharge data and validated from procedural reports. Exclusion criteria were insertion of a cardiac resynchronisation therapy device, implantable cardiac defibrillators, elective PPM insertions, patients admitted from RACF, substantially incomplete or unavailable discharge summaries, or day cases transferred from non-WA health peripheral hospitals. Patients with presenting complaints unrelated to heart block were excluded.

### Data collection

Individual patients were identified using a specific statewide medical record number allocated on first public hospital admission. Arrhythmia type and device type were obtained from a local cardiology specific procedural database. Bradyarrhythmias were categorised as 3rd degree atrioventricular (AV) block, 2nd degree Mobitz I AV block, 2nd degree Mobitz II AV block, trifascicular block or sinus node dysfunction and other. Clinic letters and discharge summaries were used to corroborate these data. The electronic medical record database was used to review relevant blood test results, and electronic discharge summaries from the index admission were used to obtain information on vital signs, admission diagnoses, complications, past medical history, medications, chronotrope requirement and admission ward (intensive care unit (ICU), coronary care unit (CCU), or ward/unknown). Complications were defined as medical events occurring during the index hospitalisation that were not directly related to the PPM insertion.

### Time from admission to PPM insertion

Date and time of hospital admission was captured using the electronic medical record system. A local cardiology specific procedural database was used to obtain the date and time of PPM insertion and the duration from admission to pacemaker insertion was thus calculated. This variable was examined as a continuous variable (days) and also dichotomised into early (< 48 h) and delayed (≥48 h), which is exploratory, but represents a practical threshold in clinical practice and is the approximate median duration of time from admission to PPM insertion.

### Discharge destination

Patients were followed up from PPM insertion until meeting the primary endpoint or censoring at 31st December 2025. The primary endpoint was death at follow-up, and the co-primary endpoint was RACF admission at follow-up. Patients who may have experienced both an RACF admission and death at separate timepoints would be included in analyses of both endpoints. Secondary endpoints included death and RACF admission at 12 months after PPM insertion (supplementary appendix).

RACF admission is used as a surrogate measure for functional decline, or loss of independence, and was defined as residing in a non-hospital living facility providing 24-hour nursing care [14]. Address on admission and discharge were identified from index discharge summaries, and living facility websites were reviewed to ensure they met criteria for RACF. If not discharged directly to RACF after index hospitalisation, RACF admission at follow-up was established from discharge summaries of subsequent public hospitalisations within this time frame. If this was unclear or there was no readmission, we assumed patients remained at the same residence since index discharge. Death was captured using the statewide electronic medical record system.

### Statistical analysis

Continuous variables are described as mean±standard deviation or median [25th – 75th percentile], depending on their distribution. Categorical variables are described as number (N) and percent. Mann Whitney U tests or Student t-tests were used to compare non-Gaussian and Gaussian continuous variables, respectively. Proportions were compared with the chi square test or Fisher exact test (if *N* ≤ 5). The association between time from admission to pacemaker insertion and outcomes (death or RACF admission) were assessed using a Cox proportional hazards model. The time to censoring, in those without outcomes, was the number of days from PPM insertion to the final data-analysis date of 31st December 2025.

Models were adjusted for clinically relevant covariates in subsequent models. Variables from Table 1 are included in the models as covariates, except for “duration of admission” which is likely colinear with time from admission to PPM insertion, and medications used of which the relevant ones are likely captured in medical comorbidities. The admission ward variable is coded as an ordinal variable for the model, incrementally increasing in value from ward/other, to coronary care unit (CCU), to intensive care unit (ICU). Similarly, the haemodynamic support variable is coded as none, one modality (external pacing or chronotropic support) or both modalities. To avoid complete separation in some models, arrythmia subtype was combined into three categories (Sinus node dysfunction / Other / Trifascicular block, Second degree AV block, Third degree AV block). The models were assessed for proportional hazard assumptions using Schoenfeld tests. Kaplan-Meier plots were used to illustrate the relationship between early and delayed PPM groups, and log-rank tests were used to test outcome distributions between groups. Additional analyses are presented in the supplement. The manuscript is reported in accordance with STROBE guidelines [15]. Statistical significance is defined as *p* < 0.05. All statistical analyses were performed on SPSS (IBM, version 29.0.0) and R-studio (version 2024.09.1).

**Table 1:** Baseline characteristics and admission details

*N*(%), Mean ± SD OR Median [25^th^ – 75^th^]	Whole cohort	<48 h between admission and PPM insertion (*n*=194)	≥48 h between admission and PPM insertion (*n*=181)	*P*-value
*Demographics*				
Male	228 (60.8%)	117 (59.3%)	111 (61.3%)	0.84
Age (years)	81±7	81±8	81±7	0.99
*Past Medical History*				
Atherosclerotic CVD	170 (45.3%)	89 (45.9%)	81 (44.8%)	0.83
CCF	63 (16.8%)	33 (17.0%)	30 (16.6%)	0.91
COPD	54 (14.4%)	25 (12.9%)	29 (16.0%)	0.39
Malignancy	72 (19.2%)	36 (18.6%)	36 (19.9%)	0.74
Cirrhosis	5 (1.3%)	3 (1.5%)	2 (1.1%)	1.00
Diabetes Mellitus	113 (30.1%)	60 (30.9%)	53 (29.3%)	0.73
Cognitive Impairment	43 (11.5%)	18 (9.3%)	25 (13.8%)	0.17
Parkinson’s syndrome	8 (2.1%)	5 (2.6%)	3 (1.7%)	0.73
Renal Failure	62 (16.5%)	30 (15.5%)	32 (17.7%)	0.56
Visual Impairment	23 (6.1%)	12 (6.2%)	11 (6.1%)	0.97
*Medications on Admission*				
Any antihypertensive	299 (79.7%)	153 (78.9%)	146 (80.7%)	0.67
Beta blocker	118 (31.5%)	67 (34.5%)	51 (28.2%)	0.19
Anticoagulant	102 (27.2%)	50 (27.7%)	52 (28.8%)	0.50
Antipsychotic	8 (2.1%)	4 (2.1%)	4 (2.2%)	1.00
PPI	144 (38.4%)	79 (40.7%)	65 (35.9%)	0.34
*Admission Tests*				
Haemoglobin (g/L) (*n*=373)	128±21	130±21	127±22	0.21
Neutrophils (x10^9^/L) (*n*=373)	5.48 [4.02-7.57]	5.08 [3.97-6.62]	5.80 [4.25-8.41]	0.002
*Arrhythmia type*				
Sinus node dysfunction / other	185 (49.3%)	76 (39.2%)	109 (60.2%)	<0.001
Trifascicular block	12 (3.2%)	3 (1.5%)	9 (5.0%)	0.08
Third degree AV block	123 (32.8%)	85 (43.8%)	38 (21.0%)	<0.001
Mobitz I second degree AV block	10 (2.7%)	3 (1.5%)	7 (3.9%)	0.21
Mobitz II second degree AV block	45 (12.0%)	27 (13.9%)	18 (9.9%)	0.24
*Haemodynamic support*				
Chronotrope Use	74 (19.7%)	53 (27.3%)	21 (11.6%)	<0.001
External Pacing	10 (2.7%)	5 (2.6%)	5 (2.8%)	1.00
*Admission ward*				
ICU	6 (1.6%)	2 (1.0%)	4 (2.2%)	0.44
CCU	80 (21.3%)	57 (29.4%)	23 (12.7%)	<0.001
Ward / Unknown	289 (77.1%)	135 (69.6%)	154 (85.1%)	<0.001
*Other*				
Duration of Admission (Days)	3.86 [2.15-6.24]	2.31 [1.79-3.22]	5.96 [4.16-9.27]	<0.001

CVD: cardiovascular disease, CCF: congestive cardiac failure, COPD: Chronic obstructive pulmonary disease, PPI: proton pump inhibitor, AV: atrioventricular, ICU: intensive care unit, CCU: coronary care unit

## Results

### Baseline characteristics

**Fig. 1:**
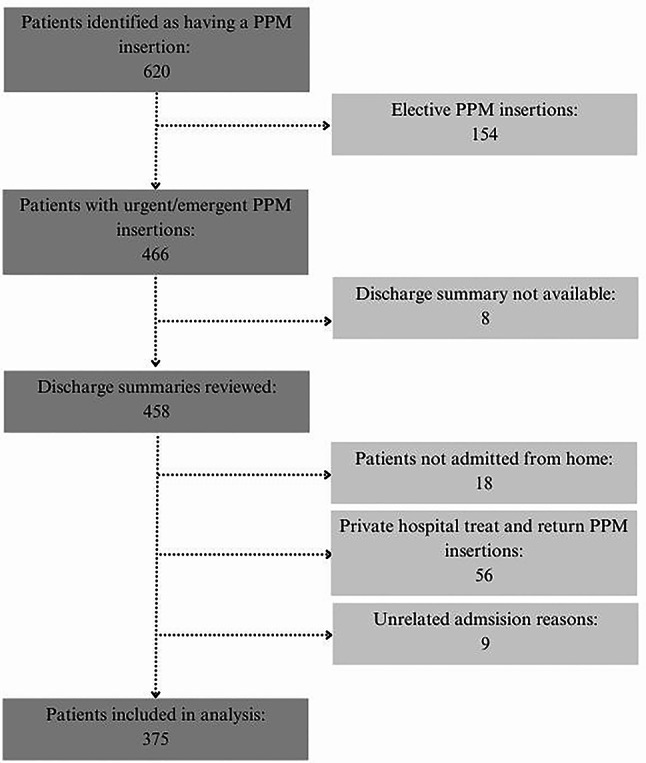
CONSORT Flow chart of patients included in the study. The figure demonstrates the number of patients screened, discharge summaries reviewed, and included in the current analysis. Exclusion reasons are described

In total, 620 patients were screened, and 375 were eligible and included in the final analysis (Fig. 1). The mean age of the included cohort was 80.8±7.5 and 228 (60.8%) were male. Comorbidities were common and medication usage was frequent (Table 1). The most common indication for PPM insertion was sinus node dysfunction or other (*N* = 185, 49.3%), followed by 3rd degree atrioventricular (AV) block (*N* = 123, 32.8%).

### Characteristics of patients with early or delayed PPM insertion

The median time from admission to PPM insertion was 47.0 [21.8–90.1] hours or 2.0 [0.9–3.8] days. There were 181 (48.3%) patients with delayed (≥48 h) PPM insertion. Compared to patients with early (< 48 h) PPM insertion, patients with delayed PPM insertion had a lower prevalence of 3rd degree AV block (21.0% v 43.8%, *p* < 0.001), higher prevalence of sinus node dysfunction / other arrythmia (60.2% v 39.2%, *p* < 0.001), lower prevalence of chronotrope use (11.6% v 27.3%, *p* < 0.001), higher neutrophil count (5.80 [4.25–8.41] x10^9^/L v 5.08 [3.97–6.62] x10^9^/L, *p* = 0.002), lower prevalence of CCU admission (12.7% v 29.4%, *p* < 0.001), and higher prevalence of ward admission (or unknown admission ward) (85.1% v 69.6%, *p* < 0.001) (Table 1).

### Relationship between time from admission to PPM insertion and death

Patients were followed-up for a median of 1635 [1048–1931] days until death or censoring. During this time, 163 (43.5%) patients died. Death occurred in 50.3% (91/181) of the delayed PPM insertion group compared with 37.1% (72/194) in the early PPM insertion group, resulting in an absolute risk difference of 13.2% and a number needed to harm of 8.

Time from admission to PPM insertion (days) was associated with an increased risk of death at follow-up in a univariate regression model (HR 1.05, 95% CI [1.03–1.07], *p* < 0.001) and after sequential adjustment for demographics, comorbidities, presenting characteristics and number of inpatient complications (HR 1.03 [1.00–1.05], *p* = 0.027) (Table 2). When using a dichotomous threshold for time from admission to PPM insertion, delayed PPM insertion (≥ 48 h) remained independently associated an increased risk of death (HR 1.77 [1.23–2.54], *p* = 0.002) (supplementary Table 1).

**Table 2:** Results of a Cox-proportional hazard model examining the relationship between time from admission to PPM insertion and death or RACF admission

Univariate	Death	RACF admission
	1.05 [1.03–1.07], *p* < 0.001	1.03 [1.00–1.07], *p* = 0.049
Age and Sex	1.04 [1.02–1.06], *p* < 0.001	1.03 [0.99–1.06], *p* = 0.07
+ Number of comorbidities	1.03 [1.01–1.06], *p* < 0.001	1.03 [0.99–1.06], *p* = 0.12
+ Admission tests	1.03 [1.01–1.05], *p* = 0.010	1.02 [0.98–1.05], *p* = 0.39
+ Arrhythmia type	1.03 [1.01–1.05], *p* = 0.010	1.02 [0.98–1.05], *p* = 0.36
+ Admission ward and Haemodynamic support	1.03 [1.01–1.05], *p* = 0.013	1.02 [0.98–1.05], *p* = 0.35
+ Number of inpatient complications	1.03 [1.00–1.05], *p* = 0.027	1.00 [0.96–1.04], *p* = 0.92

The table presents the results of a cox regression model testing the relationship between time from admission to PPM insertion (exposure variable, in days, as a continuous variable) and risk of death, or RACF admission, at follow-up. A univariate model is first presented, and subsequent models incrementally include the listed covariates which are obtained from Table 1. Hazard ratios, 95% Confidence intervals and p-values for the exposure variable are reported. Admission tests include Haemoglobin and Neutrophils and two patients without admission blood tests are excluded from models including these covariates

**Fig. 2:**
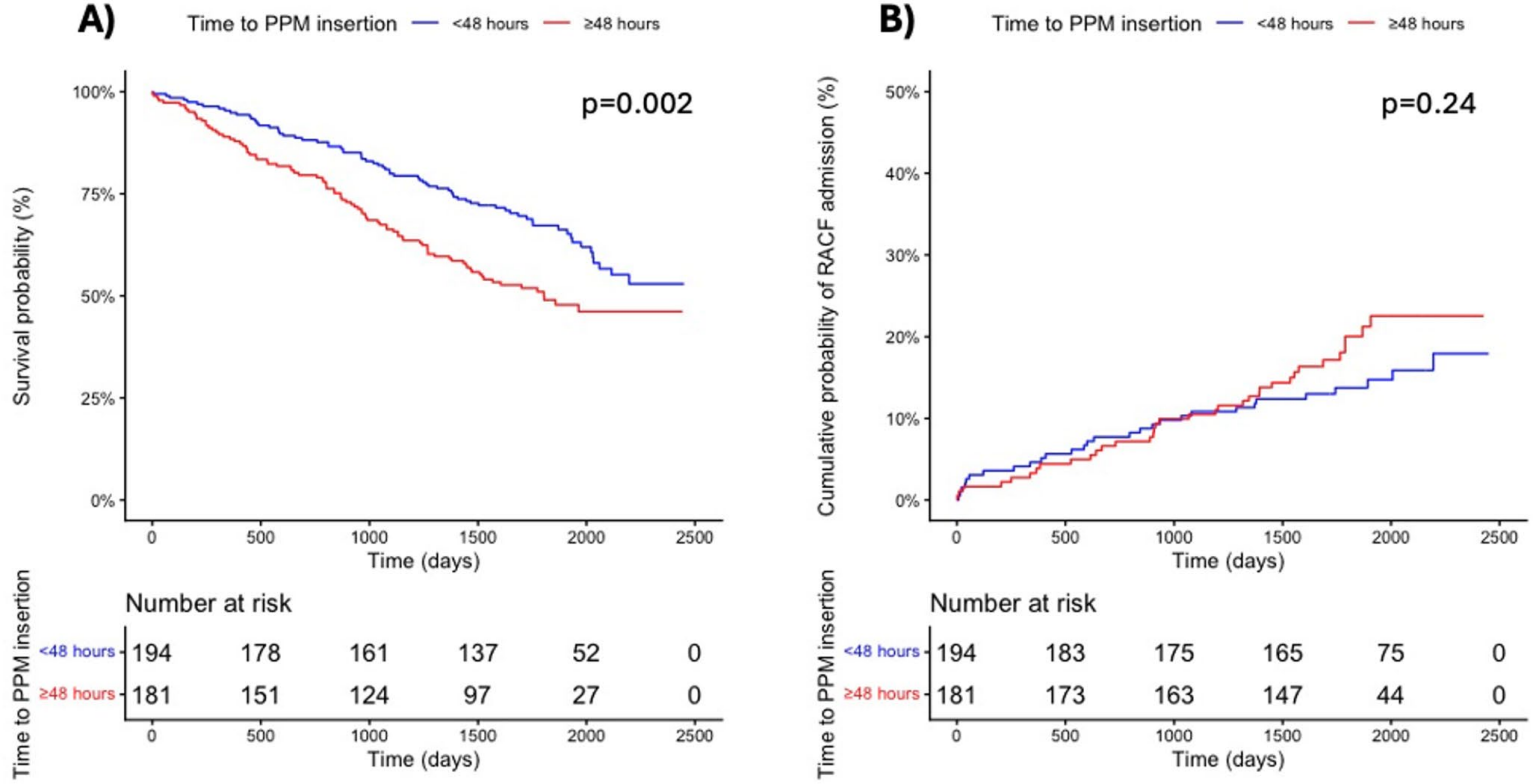
Kaplan-Meier plots demonstrating the relationship between delayed PPM insertion (≥ 48 h) and mortality, or RACF admission. Panel **A** demonstrates a Kaplan-meier survival plot with number at risk table for patients with delayed (≥ 48 h) PPM insertion, or early PPM (< 48 h) insertion. The p-value is the result of a log-rank test. Panel **B** demonstrates a Kaplan-meier cumulative probability plot with number at risk table for patients with delayed (≥ 48 h) PPM insertion, or early PPM (< 48 h) insertion. The p-value is the result of a log-rank test. Note the difference in Y-axis scales between plots

These findings were corroborated by an early diverging time-associated risk of death between early and delayed PPM-insertion groups in Kaplan-Meier survival plots (log-rank p-value 0.002, Fig. 2a). A similar association was seen when examining time from admission to PPM insertion as three groups (≤ 2 days, 2–5 days, ≥ 5 days, log-rank p-value 0.004, supplementary Fig. 1a).

### Relationship between time from admission to PPM insertion and RACF admission

Patients were followed-up for a median of 1789 [1546–2128] days until RACF admission or censoring. During this time, 64 (17.1%) patients were admitted to an RACF. RACF admission occurred in 19.3% (35/181) of the delayed PPM insertion group compared with 14.9% (29/194) in the early PPM insertion group, resulting an absolute risk difference of 4.4% and a number needed to harm of 23.

Time from admission to PPM insertion (days) was associated with an increased risk of RACF admission at follow-up in a univariate regression model (HR 1.03 [1.00–1.07], *p* = 0.049), but not in multivariable modelling (Table 2). There was no association between time from admission to PPM insertion and RACF admission when time was analysed as a dichotomous variable (supplementary Table 1).

There was no significant time-associated risk of RACF admission between early and delayed PPM insertion groups in Kaplan-Meier cumulative probability plots (log-rank p-value 0.24, Fig. 2b), nor when examining time from admission to PPM insertion as three groups (log-rank p-value 0.27, supplemental Fig. 1b). However, a delayed divergence in probability of RACF admission is observed after approximately 1500 days (Fig. 2b, supplemental Fig. 1b).

### Relationship between time from admission to PPM insertion and death or RACF admission at 12-months

In total, 31 patients died by 12 months follow-up. In multivariable regression models, the association between time from admission to PPM and death at 12-months persisted when analysed as a dichotomous variable (delayed PPM insertion HR 3.07 [1.29–7.32], *p* = 0.011, supplemental Table 2), but not when analysed as a continuous variable (HR 1.04 [0.99–1.10], *p* = 0.12, supplemental Table 3).

In total, 16 patients had been admitted to an RACF at 12-month follow-up. In multivariable regression models, there was no association between time from admission to PPM insertion and RACF admission at 12-months when examining time from admission to PPM insertion as a dichotomous variable (HR 0.62 [0.20–1.91], *p* = 0.41, supplemental Table 2), or as a continuous variable (HR 0.96 [0.90–1.02], *p* = 0.20, supplemental Table 3).

### Within hospital complications of delayed PPM insertion

We explored the relationship between delayed PPM insertion and within hospital complications (Table 3). Those with a delayed PPM insertion, compared to those without, had a higher prevalence of infectious complications (14.4% v 7.2%, *p* = 0.025), medical emergency team (MET) response calls (8.8% v 1.5%, *p* = 0.002) and any complications (39.8% v 23.7%, *p* < 0.001). Death at follow-up was more common in patients with any complication compared to those without (61.9% v 35.0%, *p* < 0.001), in those with delirium compared to those without (84.0% v 40.6%, *p* < 0.001) and in those with infection or sepsis compared to those without (67.5% v 40.6%, *p* = 0.001). Similarly, admission to RACF was more common in patients with any complication compared to those without (23.7% v 14.4%), in those with delirium compared to those without (44.0% v 17.8%, *p* < 0.001) and in those with infection or sepsis compared to those without (35.0% v 14.9%, *p* = 0.001).

**Table 3:** Prevalence of inpatient complications occurring in patients who had early or delayed PPM insertion

In hospital adverse events	< 48 h between admission and PPM insertion (*n* = 194)	≥ 48 h between admission and PPM insertion (*n* = 181)	*P*-value
Any adverse hospital event (unrelated to PPM insertion)	46 (23.7%)	72 (39.8%)	< 0.001
AKI	18 (9.3%)	21 (11.6%)	0.46
Delirium	12 (6.2%)	13 (7.2%)	0.70
DVT or PE	0 (0%)	1 (0.5%)	0.48
Fall	2 (1.0%)	3 (1.7%)	0.68
Infection or Sepsis	14 (7.2%)	26 (14.4%)	0.025
MET call	3 (1.5%)	16 (8.8%)	0.002

The table demonstrates the prevalence of inpatient complications between groups of patients with and without delayed PPM insertion. AKI: acute kidney injury, DVT: deep vein thrombosis, PE: pulmonary embolism, MET: medical emergency team

## Discussion

In this retrospective cohort study of older persons undergoing urgent or emergent PPM insertion, we observed an increased risk of death with increasing time from admission to PPM insertion. This relationship existed despite the increased acuity of patients with early PPM insertion and persisted after comprehensive adjustment for multiple clinically relevant covariates. We also demonstrated increased risk of RACF placement with increasing time from admission to PPM insertion, although this relationship was attenuated after multivariable adjustment. Finally, we identified an association between delayed PPM insertion and the risk of inpatient complications, and an association between inpatient complications and downstream death or RACF admission. The uniquely older adult population studied and long follow-up provides novel and comprehensive insights into the clinical importance of delays to PPM insertions.

We observe an association between longer time from admission to PPM insertion and mortality, that persisted up to 4.5 years after PPM insertion. This relationship largely persisted when time to PPM insertion was examined as a dichotomous variable, or when death was examined at 12-months. It is expected that excess mortality associated with delayed PPM insertion emerges gradually, because harms of delay likely operate through cumulative, non‑immediate mechanisms such as infection, delirium, deconditioning, and loss of physiological reserve, however these mechanisms were not directly tested in this study. Furthermore, the potential for confounding by indication and absence of baseline measures of frailty or functional status must be considered when interpreting our observations. Delays to PPM insertion have been associated with poor outcomes in other cohorts. In a retrospective study of acute pacemaker insertions in 259 patients of all ages in Denmark, waiting periods of longer than 1 day were associated with increased pre-procedure adverse events compared to 1 day or less (36.7% v 20.4%, *p* < 0.05) [16]. The most common complication overall was asystole (defined as a pause lasting > 3.5s), but in those with a wait time of 6 days or more, infection was the most common complication [16]. However, longer term outcomes were not assessed in the study. In a separate retrospective cohort study of older persons (aged 80 years or older, *N* = 100), 90% were alive at 1 year post PPM insertion but a longer length of stay prior to PPM insertion was associated with a higher risk of all-cause mortality (HR 1.03 [1.02–1.05] per day, *p* < 0.001) [17]. These observations are similar to our study, however, we expand the population to include all patients aged ≥ 65 years old (those eligible for RACF admission in Australia) and we include RACF specific endpoints representative of a treatment goal for PPM insertion: maintenance of functional independence.

There was a small increased risk of RACF admission with increasing delays to PPM insertion that was present on univariate regression models and was attenuated after adjustment for relevant covariates. Therefore, whilst an association between delayed PPM insertion, deconditioning, and poorer functional outcomes is logical, it was not observed in this cohort. However, a late divergence observed in cumulative probability plots raises the possibility of a delayed effect of delayed PPM insertion on RACF admission. Entry into RACF often reflects a cumulative functional decline influenced by prolonged recovery trajectories, caregiver capacity, and delays in access to RACF. For example, the median time to receive Australian government supported RACF care after application is 267 [186–457] days [18]. In our study, an RACF-listed address on a hospital discharge summary (either on discharge from index admission or on a subsequent admission) was required to validate the RACF endpoint and this, combined with expected delays to RACF admission in Australia, may partially explain the absence of a significant association between delayed PPM insertion and RACF admission seen in our study. Functional limitations of activities of daily living are a common reason for RACF admission [19]. Patients with bradycardia, particularly if symptomatic, are often given instructions for bed rest [20]. Even post PPM insertion, restrictions may be placed on arm movement [21]. Delays to PPM insertion may be associated with longer bed rest, and associated deconditioning can occur within two days of admission [22]. In a study of post-surgical older adults, 59.6% of patients experienced some loss of independence (defined as a decline in functional status, mobility, and/or care needs) and this was independently associated with death after discharge (OR 6.7 [2.4–19.3]) [23]. Future studies examining the functional effects of delays to PPM insertion using of quantitative metrics of mobility or frailty, are needed.

In our study, non-pacemaker related inpatient complications, notably infections, were more prevalent in patients waiting at least 48 h for PPM insertion. Notably, this was an exploratory endpoint in our study, and it is not possible to accurately determine if infections occurred before or after PPM insertion, and a causal relationship between delays to PPM insertion and infectious complications cannot be presumed. We also observed that patients with infectious complications were more likely to enter an RACF or be deceased at follow-up. Taken together, these findings suggest that either delays to PPM insertions are associated with increased risk of infectious complications, or that infections may contribute to delays to PPM insertion, and either pathway associates with increased risk of poorer outcomes at follow-up. A Brazilian study of 905 patients observed that up to 10% of patients developed hospital acquired infections whilst awaiting PPM insertion. Those with pre-PPM infections were associated with longer median wait times (17 [13, 24] v 4 [2, 8] days, *p* < 0.001) and median length of hospital stay (21 [14, 31] v 6 [3, 10], *p* < 0.001). Hospital acquired infections prior to insertion of a PPM were also associated with greater in hospital mortality (5.6% v 1.6%, *p* = 0.01) and 30 day mortality (7.9% v 2.1%, *p* = 0.001) [24]. We also observed a heightened prevalence of delirium in patients who later died or were admitted to an RACF, though this complication was not associated with delay to PPM insertion. Other unexplored factors may contribute to risk of poor outcomes in older adults requiring PPM insertion.

Our observations provide insight into potential pathways for improving the care and outcomes of older adults admitted for urgent or emergent PPM insertion. Addressing delays to PPM insertion may be a feasible approach to reduce the risk of inpatient complications, deconditioning, RACF admission or death. Furthermore, fewer delays to PPM insertion may reduce duration of admission, which may translate to reduced total healthcare costs for care providers and hospitals [25]. Whilst it may not be feasible to prevent some delay in all settings, the current data and logic suggest substantial delays to PPM would best be minimised, and strategies to prevent complications associated with delayed PPM insertion may be beneficial when delay occurs. Prehabilitation for older adults has been explored in surgical settings and found to minimise post-operative deconditioning [26]. This can be explored with modification for patients awaiting PPM; low resistance bed-based exercises being found to improve sit to stand strength in older adults bed bound for at least 1 month [27, 28]. Nutritional supplementation may also be beneficial, as it is in poorly nourished surgical patients [29]. Finally, best-clinical practice dictates that avoidance of other risk factors such as unnecessary indwelling catheters or intravenous cannulas is important to reduce the risk of infection [24]. Overall, these observations also highlight areas for future research, including administrative and policy-making processes, staffing models, 24/7 service provision, access to surgical or catheter laboratories, case prioritisation in busy clinical settings. However, prospective studies are needed before practice-changing recommendations can be made.

This study has several limitations. The retrospective observational nature of this study precludes the ability to determine a causal relationship between delayed PPM insertion and RACF placement or death. Despite comprehensive regression modelling, the effect of residual confounding remains probable, particularly from unmeasured factors such as frailty, poorly captured medical comorbidities or unaccounted for illness at time of presentation. This study was conducted in a single-centre with weekday procedural availability and on-call weekend availability, and only older adults were included. This may preclude generalisability to other populations of other ages, or to other sites that have on-call only or 24/7 services. RACF admission has been considered a surrogate for significant deconditioning, or loss of functional independence, however it is acknowledged that patients may enter an RACF for other reasons including chronic illness, psychosocial needs or caregiver availability, and this may result in misclassification of the co-primary outcome. Deconditioning, whilst hypothesised to be on the causal pathway between delayed PPM insertion and RACF admission or death, could not be reliably determined from these retrospective data. Furthermore, other medical conditions such as delirium are obtained from medical discharge summaries, not standardised scoring tools. It is possible that RACF placement and mortality are under-reported, due to subsequent private hospital admissions or direct from home-to-RACF admissions not captured by our analyses. Death was identified using electronic medical records, which may not capture all cases of death in the community or outside of the public health system. Delays to PPM may be due to medical conditions that were not temporally captured in our data analysis and due to the inability to temporally describe inpatient complications, it is not possible to ascertain whether these conditions may have existed before PPM insertion, at least partially.

## Conclusion

In this observational study of older adults requiring urgent or emergent PPM insertion, longer times from admission to PPM insertion was associated with higher mortality over a median follow-up of 4.5 years. Delays to PPM insertions were not associated with a greater risk of RACF admission over 4.9 years. Delays to PPM insertion were associated with more in-hospital complications, including infection. Future prospective research is needed to determine if reductions in time to PPM insertion may improve health outcomes amongst older adults.

## Supplementary Material

Below is the link to the electronic supplementary material.

Supplementary Material 1

## Data Availability

Data can be made available upon reasonable request to the corresponding author.
