# Supplementary material for: The relationship between time to urgent or emergency pacemaker implantation and entry into residential aged care or death in older adults
Source: Aging Clin Exp Res. 2026 Jun 7;38(1):177. doi: 10.1007/s40520-026-03413-4 (PMC13478027; doi:10.1007/s40520-026-03413-4)
Supplement: Supplementary file 1 [file 40520_2026_3413_MOESM1_ESM.docx]

**Supplementary appendix for: The relationship between time to urgent or emergency pacemaker implantation and entry into residential aged care or death in older adults**

Diane Nguyen (0009-0007-8367-6290)^1^, Madeleine Landwehr^2^, Caitlyn Taylor^1^, Eng Sim^1^, Stephen Bacchi (0000-0001-5130-8628)^3^, Graham S. Hillis (0000-0003-2417-4673)^4,5^, Leon Flicker (0000-0002-3650-0475)^1,5^, Carl Schultz (0000-0002-0847-4361)^4,5^, Lucy Kilshaw^1^, Nikola Stoyanov (0009-0005-4588-3526)^4^, Jamie W. Bellinge (0000-0001-8257-7361)^5^

1. Department of Geriatrics, Royal Perth Hospital, Perth, Western Australia, Australia
2. Department of Psychiatry, Royal Perth Hospital, Perth, Western Australia, Australia
3. Faculty of Health and Medical Sciences, University of Adelaide, Adelaide, South Australia, Australia
4. Department of Cardiology, Royal Perth Hospital, Perth, Western Australia, Australia
5. Medical School, University of Western Australia, Perth, Western Australia, Australia

Corresponding author:

Dr Jamie W Bellinge

Medical School

University of Western Australia

35 Stirling Highway

Crawley, Perth, Western Australia

Australia, 6009

[Jamie.Bellinge@uwa.edu.au](mailto:Jamie.Bellinge@uwa.edu.au)

**Supplemental Table 1.** Results of a Cox-proportional hazard model examining the relationship between time from admission to PPM insertion, and death or RACF admission

|  | **Death** | **RACF admission** |
| --- | --- | --- |
| **Univariate** | 1.62 [1.19 – 2.21], p=0.002 | 1.34 [0.82 – 2.20], p=0.24 |
| **Age and Sex** | 1.75 [1.28 – 2.39], p<0.001 | 1.35 [0.83 – 2.22], p=0.23 |
| **+ Number of comorbidities** | 1.83 [1.33 – 2.50], p<0.001 | 1.35 [0.83 – 2.22], p=0.23 |
| **+ Admission tests** | 1.67 [1.21 – 2.30], p=0.002 | 1.17 [0.70 – 1.95], p=0.55 |
| **+ Arrhythmia type** | 1.77 [1.25 – 2.50], p=0.001 | 1.27 [0.75 – 2.14], p=0.38 |
| **+ Admission ward and haemodynamic support** | 1.78 [1.25 – 2.54], p=0.001 | 1.19 [0.70 – 2.02], p=0.52 |
| **+ Number of inpatient complications** | 1.77 [1.23 – 2.54], p=0.002 | 1.07 [0.62 – 1.85], p=0.81 |

The regression model demonstrates the results of Cox proportional hazards models testing the relationship between time from admission to PPM insertion (<48 hours vs ≥48 hours) and risk of death or RACF admission at follow-up. A univariate model is first presented, and subsequent models incrementally include the listed covariates. Hazard ratios and 95% confidence intervals are reported. Two patients without admission blood tests were excluded.

**Supplemental Table 2.** Results of Cox-proportional hazard models examining the relationship between time from admission to PPM insertion, and 12-month mortality or RACF admission

|  | **12-month Death** | **12-month RACF admission** |
| --- | --- | --- |
| **Univariate** | 2.33 [1.10 – 4.95], p=0.028 | 0.83 [0.31 – 2.22], p=0.71 |
| **Age and Sex** | 2.34 [1.10 – 4.98], p=0.027 | 0.82 [0.31 – 2.21], p=0.70 |
| **+ Number of comorbidities** | 2.23 [1.05 – 4.75], p=0.038 | 0.74 [0.27 – 2.01], p=0.56 |
| **+ Admission tests** | 2.14 [0.99 – 4.63], p=0.052 | 0.72 [0.26 – 2.03], p=0.54 |
| **+ Arrhythmia type** | 2.88 [1.26 – 6.62], p=0.012 | 0.78 [0.27 – 2.26], p=0.65 |
| **+ Admission ward and haemodynamic support** | 2.99 [1.26 – 7.10], p=0.012 | 0.74 [0.25 – 2.21], p=0.59 |
| **+ Number of inpatient complications** | 3.07 [1.29 – 7.32], p=0.011 | 0.62 [0.20 – 1.91], p=0.41 |

The table demonstrates the results of Cox proportional hazards models testing the relationship between time from admission to PPM insertion ≥48 hours (compared with <48 hours) and risk of death or RACF admission within 12 months. A univariate model is first presented, and subsequent models incrementally include the listed covariates. Hazard ratios and 95% confidence intervals are reported. Two patients without admission blood tests were excluded from adjusted analyses.

**Supplemental Table 3.** Results of Cox-proportional hazard models examining the relationship between time from admission to PPM insertion and 12-month mortality or RACF admission

|  | **12-month Death** | **12-month RACF admission** |
| --- | --- | --- |
| **Univariate** | 1.04 [1.00 – 1.08], p=0.045 | 1.04 [0.98 – 1.10], p=0.16 |
| **Age and Sex** | 1.04 [1.00 – 1.08], p=0.07 | 1.03 [0.98 – 1.09], p=0.25 |
| **+ Number of comorbidities** | 1.02 [0.98 – 1.07], p=0.28 | 1.02 [0.96 – 1.08], p=0.53 |
| **+ Admission tests** | 1.02 [0.98 – 1.07], p=0.40 | 1.02 [0.96 – 1.08], p=0.55 |
| **+ Arrhythmia type** | 1.04 [0.99 – 1.09], p=0.15 | 1.00 [0.95 – 1.06], p=0.92 |
| **+ Admission ward and haemodynamic support** | 1.03 [p=0.99 – 1.08], p=0.17 | 1.00 [0.95 – 1.06], p=0.95 |
| **+ Number of inpatient complications** | 1.04 [0.99 – 1.10], p=0.12 | 0.96 [0.90 – 102] , p=0.20 |

The regression model demonstrates the results of Cox proportional hazards models testing the relationship between time from admission to PPM insertion (days) and risk of death or RACF admission within 12 months. A univariate model is first presented, and subsequent models incrementally include the listed covariates. Hazard ratios and 95% confidence intervals are reported. Two patients without admission blood tests were excluded from adjusted analyses.

**Supplementary figure 1.** Kaplan-Meier plots demonstrating the relationship between delayed PPM insertion groups and mortaltiy, or RACF admission.

Panel A demonstrates a Kaplan-meier survival plot with number at risk table for patients with differing times from admission to PPM insertion (≤2 days, 2-5 days and ≥5 days). The p-value is the result of a log-rank test. Panel B demonstrates Kaplan-meier cumulative probability for RACF admission plot with number at risk table for patients with differing times from admission to PPM insertion (≤2 days, 2-5 days and ≥5 days). The p-value is the result of a log-rank test. Note the difference in Y-axis scales between plots.
